# Integration of a surgical robotic arm to the connected operating room via ISO IEEE 11073 SDC

**DOI:** 10.1007/s11548-023-02926-x

**Published:** 2023-05-13

**Authors:** Noah Wickel, Manuel Vossel, Okan Yilmaz, Klaus Radermacher, Armin Janß

**Affiliations:** Chair of Medical Engineering, RWTH Aachen University, Aachen, Germany

**Keywords:** ISO IEEE 11073 SDC, Operating room network, Surgical robotics, Interoperability, DevSpecs, Plug-and-Play

## Abstract

**Purpose:**

Since 2019, intraoperative networking with ISO IEEE 11073 SDC has, for the first time, enabled standardized multi-vendor data exchange between medical devices. For seamless plug-and-play integration of devices without previous configuration, further specifications for device profiles (“device specializations”) on top of the existing core standards must be developed. These generic interfaces are then incorporated into the standardization process.

**Methods:**

An existing classification scheme of robotic assistance functions is being adopted and used as a baseline to derive functional requirements for a universal interface for modular robot arms. Additionally, the robot system requires machine-machine interfaces (MMI) to a surgical navigation system and a surgical planning software in order to carry out its function. Further technical requirements are derived from these MMI. The functional and technical requirements motivate the design of an SDC-compatible device profile. The device profile is then assessed for feasibility.

**Results:**

We present a new modeling of a device profile for surgical robotic arms intended for neurosurgery and orthopedic surgery. The modeling in SDC succeeds for the most part. However, some details of the proposed model cannot yet be realized within the framework of the existing SDC standards. Some aspects can already be realized, but could be better supported in the future by the nomenclature system. These improvements are being presented as well.

**Conclusion:**

The proposed device profile presents a first step toward a uniform technical description model for modular surgical robot systems. The current SDC core standards lack some functionality to fully support the proposed device profile. These could be defined in future work and then included in standardization efforts.

## Introduction

In recent years, a multitude of different systems by various manufacturers have been developed and marketed in the field of surgical robotics. Their aim is mostly to relieve the clinical staff and enhance surgical precision for critical procedures. Examples in the disciplines of orthopedic surgery and neurosurgery include the placement of spinal pedicle screws, bone sawing for implant insertion or stereotactic biopsy [1]. The aim is the accurate execution of a pre- or intraoperative surgical plan and the elimination of human error. Minimally invasive approaches are facilitated [2]. The robot arm is used as a positioning aid for passive and active surgical tools such as drills, taps, screwdrivers, burrs or needles. Examples of such systems are Excelsius GPS (Globus Medical), Mazor X Stealth (Medtronic) and ROSA Spine/Brain (Zimmer Biomet).

Among the advantages of surgical robotics are increased accuracy and shorter X-ray exposition time [2, 3]. However, these findings are subject to debate due to various associated disadvantages. These include high initial costs, limited clinical applications and prolonged pre- and intraoperative time due to deficient usability [4, 5]. The systems are marketed as all-in-one solutions and often lack a suitable software interface to enable interoperability to devices and software of other manufacturers, creating lock-in effects and preventing the efficient inclusion of specialized devices of small and medium-sized enterprises (SME) into the procedure. The workflows cannot be unified through central user interfaces.

At the same time, robot arms exhibit a high degree of flexibility. Their scope of application could extend beyond the few current surgical tasks, instead being reused modularly for a wide range of applications. The systems considered in the present work all serve a critical positioning task, executed with the help of an (optical) tracking system according to a surgical plan. The desired plug-and-play compatibility between the robot system and other relevant devices requires the standardization of a generic, open interface, supported by multiple vendors and implemented in their products. Hence, the intended robot interface is not a specific description of one device’s functionality, but rather a universally applicable gateway derived from a generic device profile. Using such an interface, the components of the robotic surgery solution (including the robot arm and base, the optical or electromagnetic tracking, the surgical planning software, implant-specific tools and the intraoperative user interface and visualization) could be modularized and then sourced and replaced independently, flexibly combined or partially mounted permanently into the operating room (OR). Ultimately, the resulting flexibility and usability could shorten operating times, reduce expenses and improve the clinical outcome for patients.

The present work showcases such an interoperable device profile and discusses the pros and cons of the resulting model. Initially, an overview of the ISO IEEE 11073 SDC core standards and an example for the clinical use of robot arms in the operating room is given. The following section discusses a selection of related works by other authors before the methodology and influences for the new robot device profile are portrayed and the resulting SDC model is presented. Finally, the section “Technical limitations & improvements” provides an evaluation of the identified shortcomings of SDC for robot modeling and proposes improvements and future work.

## Background

### Medical device modeling in SDC

The ISO IEEE 11073 SDC core standards [6–8] allow for safe transmission of medical parameters and signals between medical devices, displays and input devices of various manufacturers. SDC is therefore disrupting existing monopolies in the market created by proprietary all-in-one OR systems. It enables more flexible compilation of device ensembles with the best suited components for a specific medical intervention. Hospital operators are able to act more flexibly and with economic independence. Unified, clearly structured user interfaces can be created which are more usable, safe and intuitive. Devices can discover each other on the network without further configuration (“plug-and-play”) and exchange data after appropriate authorization.

The core standards of the ISO IEEE 11073 SDC family (11073–20702, −10207 and −20701) enable intraoperative data exchange based on device models in a provider-consumer architecture [9]. Providers are offering parameters to the network, whereas consumer devices read, subscribe and request adjustments to parameters in accordance with the permitted operations. The provider represents itself as a rooted tree graph of height four (named medical device description, *MdDescription,* see Fig. 4). Each node can be associated with one dynamic state element (listed in a structure named *MdState*) though unique string identifiers (*handle*). The tree represents the internal structure of the medical device from coarse to fine granularity on four levels: Medical device (MDS, 1st level), virtual medical device (VMD, 2nd layer), channel (3rd layer) and metric (4th layer). *MdDescription* and *MdState* add up to the *Medical Data Information Base (MDIB)*. Designing an *MDIB*, and especially the *MdDescription* part, is a core activity of SDC device modeling.

The “leaves” of the *MdDescription* (*metrics)* represent a single parameter or physical measured variable (e.g., blood pressure) or a setting (e.g., power of a bipolar forceps). *Metrics* can be of any of the following types: *Numeric* (floating point number), *String*, *EnumString*, *RealTimeSampleArray* or *DistributionSampleArray*. The latter two are designed for series of values or stochastic distributions (one-dimensional).

Every node of the tree has, in addition to its value, an activation state (*ComponentActivation, CA*). The *CA* is a state machine which can be used to indicate the state of operation of a parameter, group or the whole device. CAs are relevant for the creation of a safe activation mechanism [6, 10].

By default, metrics can only be read by consumers on the network. All further interaction (for instance, changing the value or string of a metric) requires the definition of an *Operation*. In an *MDS* or *VMD*, there can exist a *service control object (SCO)* which lists all possible *Operations* within that subgraph. A manufacturer can therefore clearly define the allowed means of interaction between their device and another participant on the network.

Furthermore, there can be an *AlertSystem* attached to an *MDS* or *VMD*. The *AlertSystem* consists of *AlertConditions* and an *AlertSignals*, which are an acoustic, optical or haptic indication of the alert state. The included property *AlertConditionPriority* allows to differentiate life-threatening emergencies from less critical notifications. All alerts can be collected and delivered to applicable users by a central alert management system [11].

### Interoperability through standardized device profiles

The core standards and the nomenclature system allow semantic interoperability for single parameters. For the realization of the “plug-and-play” ideal, the device representations in their entirety must become interoperable. To this end, standardized device profiles (called *device specializations*, *DevSpecs* – IEEE P11073-1072X) are needed. *DevSpecs* include the core functionalities of a device class in a given structure, which must be considered when the MDIB of an SDC provider for a device of that class is designed [12]. A device manufacturer complying with the *DevSpec* may rest assured that the device is correctly recognized by other compatible devices in the ensemble, and that communication between these devices requires no previous configuration.

These device profile templates must be designed with great care in order to represent current as well as future devices adequately. Yet, they must be specific enough to be expressive and allow the control of all relevant functions. Otherwise, they are not able to fulfill their purpose and may be neglected by the device manufacturers. Ideally, such a device profile could include (likewise standardized) component templates for frequently found subsystems. These are called *modular specializations* (*ModSpecs*), for instance a gas supply or an external control unit [9].

### Robotic systems in orthopedic surgery and neurosurgery

Common applications for hands-on surgical robots include pedicle screw placement (e.g., in scoliosis treatment or as support in spinal canal decompression), femoral & tibial resection (in total knee arthroplasty) and needle or electrode insertion (for electroencephalography, deep brain stimulation or neurosurgical biopsies).

The arm is usually mounted on a moveable cart or directly attached to the operating table. The main task of the arm for the considered surgical fields is the positioning of a guiding sleeve or active tool directly mounted to the distal end of the robot arm. Some of the tool guides may include an additional degree of freedom not controlled by the robot (for instance allowing free movement of the tool within a geometric plane). Therefore, the movement of the surgical tool is confined in a way which eases the correct execution of a tapping, burring, drilling or sawing step. Meanwhile, the system hinders or inhibits unintended movements which could lead to injuries or death [1]. In contrast to fully active systems, the surgeon always remains in control when using semi-active or synergistic systems, and at least one degree of freedom remains accessible. Any motorized tool is also triggered solely by the surgeon; however, some systems may include an automatic power cut when the system detects a critical structure at risk.

### Other tasks for robotic arms

Apart from the main surgical tasks, robot arms could be utilized for a variety of less critical assistance functions, such as holding tasks. These can improve the safety and usability during any surgery. Examples include positioning and holding of endoscopic cameras [13] or medical retractors. Further applications could include sterile trays or informational displays, held and maneuvered collision-free around the situs by a robot arm and only taking up space when needed. Likewise, robot-attached OR lights or (tracking) cameras could be conceived once robotic arms become more abundant.

### Surgical planning and localization

Surgical planning usually includes a dataset from medical imaging (computed tomography scan, *CT* or magnetic resonance imaging, *MRI*) along with additional geometric annotations such as target trajectories and critical areas to be avoided. The annotations must be made accessible to the robot system before surgery and registered with the patient during surgery. Since the target trajectories are defined in the patient coordinate system (COS), the relation to the robot COS must be determined and constantly updated by external means, such as optical or electromagnetic tracking. The relation between the reference array (tracker) on the robot and its base COS must also be known to the controller.

## Related work

A recent review by Schleer et al. [1] analyzes the human–machine-interface (HMI) and assistive functionality of surgical robot systems on the market. Two classification schemes based on related publications are presented. The first scheme considers the modes of cooperation between human and robot. The second scheme classifies robotic assistance functions. In combination, the two schemes allow for an abstract description and comparison of all considered systems. It is suggested to use the presented classification of assistance functions as a foundation for the development of generic *cooperative robotic device profiles* (*CRDP*). Simplification of compatibility and better modularity of robotic systems are motivating these profiles, as they could improve the benefit-to-cost ratio and market penetration. The classification scheme of robotic assistance functions is taken up by the present work as a foundation of requirements toward the generic robotic device profile.

Berger et al. [14] utilize the ISO IEEE 11073 SDC standard for the control of two robotic arms in a medical setup. SDC is employed to transmit the control signals between the robots and a control computer, creating a control loop through the network. Although their technical benchmark suggests acceptable latency on the isolated laboratory network, they do not consider the impact of conflicting traffic as it would occur in a real-world operating room network. Aspects of device modeling and interoperability are not discussed.

Kasparick et al. [10] show an approach for safe remote activation of critical functions. It is based on a repeated, identifiable trigger signal and includes a fallback into a safe device state when network traffic is delayed or disrupted. The approach is not specific for robotic applications, but suitable to implement some remote-control functionality such as a safety unlock signal via foot switch. It can be realized with the current SDC standards; however, there exists no mechanism to ensure that the network delay is acceptable for the use-case.

Another related work by Kasparick et al. [15] discusses the modeling of a high-frequency electrosurgical device and an associated external remote control. The presented concept for device modeling is aimed at medical devices with multiple terminals for active surgical tools, including individual configuration of these tools. Within the scope of a robotic system, this concept could be applied to the configuration of an active end-effector. The association mechanism for external control devices can be used to connect a generic foot switch unit as a safety unlock for the robot system. There is also a mention of the *modular specification* for external control devices. Generic guidelines for the design of medical device profiles could not be derived.

The publication [16] by Andersen et al. describes the SDC modeling of various medical devices for endoscopic surgery. Similar to [15], underlying concepts and generic guidelines for device modeling are not described. Instead, the models are determined through “industry consensus.” A similar discussion of robotic device modeling with leading manufacturers in the OR.NET association (www.ornet.org) would be beneficial to improve the model introduced in the present work, and to aid its progression into standardization.

Vossel et al. [17] are presenting the use of a dedicated bus system alongside the SDC network. Its purpose is the transmission of latency-sensitive device data, such as reference and measured error signals in control applications. Robotic and navigated applications would benefit from the surgical communication bus. However, the approach implies the need for a second wired connection between all medical devices which should access data on the bus. This would drastically impair the usability in clinical daily routine and complicate the implementation of plug-and-play simplicity. Although communication busses are well established in other industries such as manufacturing, they are less flexible than IP networks. The approach is therefore not considered for the present work.

A promising approach to enable real-time data traffic for SDC-enabled devices is presented by Rother et al. in [18]. The publication employs the new standard family *IEEE 802.1 Time-Sensitive Networking* (*TSN*) to delegate the traffic capacity of a given network between critical *real-time* and *best-effort traffic*. The presented software is able to automate the configuration of such a network by evaluating the *MdDescription* of connected medical devices, assessing their prospective traffic from *metric* properties. The current SDC standards cannot yet fully support all proposed features. Furthermore, specialized hardware is necessary.

## Implementation

The modeling is oriented around the following key questions:

- Which clinical functionality should be offered through the interface?
- Which structure should be chosen to present these functions?
- Which (SDC-) interfaces to other devices must be considered?

### Selection of included functionality

The main purpose of robotic systems in neurosurgery and orthopedic surgery is the positioning of a tool or a tool guide. A classification of such tasks is provided by Schleer et al. in [1], and a selected subset of these assistance functions is considered as a baseline for the design of the SDC interface. Other necessary functions for the initial setup and operation of the robot system are added. The compiled set of functional requirements is presented in Table 1.

**Table 1:** Functional requirements to the robotic SDC interface and associated assistance functions from [1]

#	Clinical function	Description	Associated assistance function
M1	Set movement mode	Safe stop, movement hands-on, or active movement toward planned target pose	
M2	Set movement constraint	Limitation of degrees of freedom (rotation only, planar translation), approach or limitation to planned trajectory or plane	Follow trajectory
M3	Set arm damping	Through setup of simulated impedance, the arm can filter human tremor during hands-on mode	Tremor reduction
M4	Set speed for active movement	Limit the speed of re-positioning when the arm moves on its own	
M5	Set collision/force limit	Arm will not continue the motion if force threshold is exceeded	Compliance control
E1	Connect to end-effector (or set passive ID)	Receive an updated coordinate transformation and mass definition for the end-effector (from endpoint or database)	
E2	Publish flange-to-end-effector transformation	Provide the coordinate transform of the currently mounted end-effector to other SDC devices (for example, a visualization)	
T1	Connect to tracking provider endpoint	Connect to the localization endpoint (tracking provider)	
T2	Receive tool pose in patient system	The tracking system updates this metric to where it currently localizes the tool pose in the patient COS	Compensation of motion
P1	Receive a surgery plan	Receive a file, string or file location with target and safety definitions in patient system	Volumetric constraints
P2	Select current target	From the many target definitions in the surgical plan, select which one is active (for approach or trajectory lock)	Find pose

### Structure of the model

**Fig. 1:**
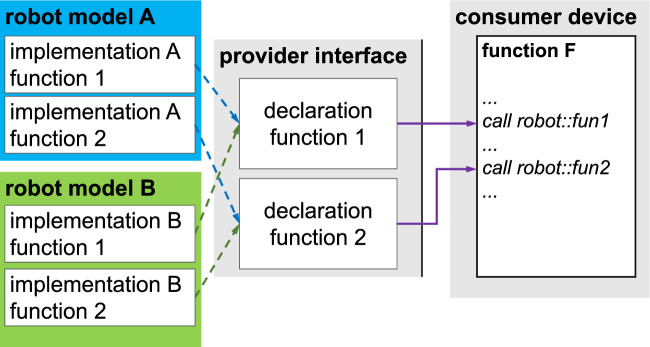
The definition of a generic interface enables uniform addressing of different implementations

When designing the device profile, it is neither intended to embed the functions of a specific clinical application, nor to translate the application programming interface (API) of the original device manufacturer into another technical language. Instead, the device profile should represent a middle ground which supports a variety of clinical applications and can be implemented through any manufacturer’s APIs, across different device variants and revisions. Therefore, it achieves interoperability (see Fig. 1).

### Interfaces to other systems

**Fig. 2:**
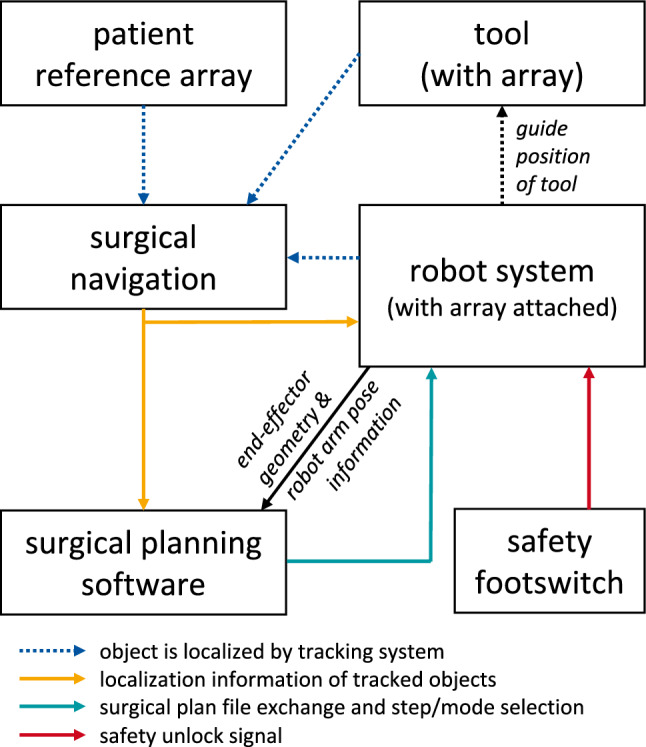
Communication paths and influences between the relevant devices during the surgery (end-effector not shown). The navigation localizes the patient, robot and surgical tool and makes this information accessible to the robot and the planning software. The robot additionally receives a surgical plan and an unlock signal from a connected device such as a foot switch

The assistance functions of the robot system require knowledge of the current pose (position + orientation) of a wielded tool relative to the patient anatomy, and furthermore the intended pose for that current step or task. Therefore, at least two different other systems must be interfaced with: A localizer (optical tracking) and a surgical planning software. Additionally, an active end-effector or tool could also exchange data with the robot. These interfaces must be considered when designing the device profile. An overview of the interactions between the system components is displayed in Fig. 2.

For the communication with the planning software, an interface for the transmission of a surgical plan and a currently intended workflow step must be available. A complex plan can include a multitude of target trajectories, planes or volumes as well as keep-out zones. For each of these annotations, at least one pose must be transmitted. For planes or cuboid volumes, two vectors are required. Arbitrarily shaped volumes can comprise point clouds made up of thousands of vectors.

The current SDC *Participant model* is inept to exchange such a plan between two endpoints. In theory, a method to transmit the plan via SDC could be conceived through misuse of existing *metric* types. However, these implementations would not conform to the intended use of the standard and would be cumbersome to use. Rather, the *Basic Intergrated Clinical Environment Protocol Standard* (“BICEPS”) employed by SDC was designed to represent the current state of clinical measurements and parameter settings. For the transmission of large amounts of structured data, we propose an approach that is based on files instead of SDC metrics (see section “Technical limitations & improvements”).

**Fig. 3:**
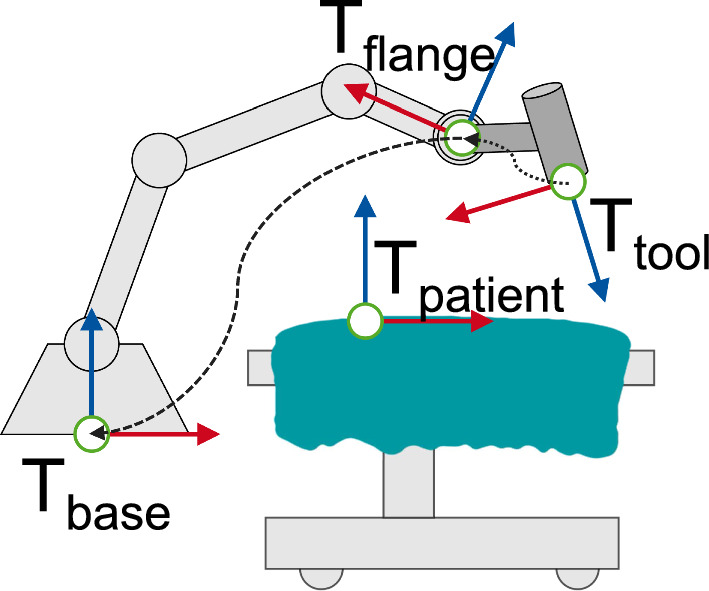
Relevant coordinate systems for the robotic application include patient, robot base, flange and tool/end-effector COS. The spatial relation between robot and patient can only be determined through external measurement by a tracking system. The dashed line represents a transformation determined by the robot. The dotted line represents the transform from flange-to-end-effector tool tip. It must be known in advance if the end-effector is passive

For the communication with a medical navigation system, the interface must support recurrent transmission of a spatial transformation between the patient COS and the robot or end-effector COS (Fig. 3). Ideally, this data is transmitted with bounded latency (real-time) to enable dynamic compensation of unintended motions, such as a ventilated patient.

Lastly, an electronic interface to the end-effector must be considered. The end-effector is the final piece in the motorized link between the robot base and the surgical tool. If a tool is permanently mounted to the robot, this tool could be considered the end-effector. Some robots are compatible with differently-shaped end-effectors for a variety of surgical procedures. Therefore, the coordinate transformation between the robot flange and the active tool tip can change, which must be communicated to the robot system.

Summarizing, the following technical requirements for the machine-machine-interface of the robotic device profile are derived from the application:

- A localization provider (for instance, optical tracking) must constantly supply the current transform between robot and patient.
- A surgical plan must be transmitted from a planning software or directory.
- The configuration of end-effector and tool must be known to the robot system before executing a movement command.
- Additionally: The current transformation between robot base and end-effector should be made available to other devices to allow visualization of the robot arm and enable collision avoidance in the case of multiple moving robots. This information is not necessarily generated by the localization provider. The robot can provide this information through measurement of its joint angles.

These technical requirements complement the functional requirements from Table 1. Together, they provide the framework for the robotic device profile presented below.

### Description of the derived device model

**Fig. 4:**
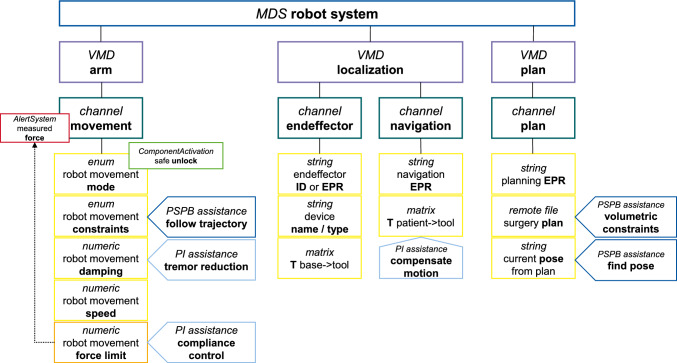
Static part of the SDC robot model (MdDescription) and realized assistance functions (blue arrows)

The chosen modeling is presented in Fig. 4. There is only a single MDS node since the robot has no detachable or otherwise separated components. Three *VMDs* are grouping settings regarding the arm, the localization and the surgical plan (including workflow control).

The *VMD* “arm” contains all the settings which directly influence the movement of the robot arm. These include changing the mode between freehand and active movement (for instance toward the selected trajectory). Settings for damping, movement speed and maximum contact force are available to cater to the required assistance functions. The contact force is realized as an alert which will stop the motion as well as notify the user when a collision appeared.

Furthermore, a metric is available to apply additional constraints to the movement of the arm in either mode. These constraints can be planning-based (trajectory, plane, volume) or planning-independent (pivot mode or translation-only). The *ComponentActivation* state of the “mode” *metric* implements a safety unlock which must be triggered according to the safe activation scheme [10]. The arm must not move unless the *CA* of this node is set to “On.”

The *VMD* “localization” manages the connection to the tracking provider and the identification of the currently mounted end-effector. An end-effector is identified either via a unique ID, or by specifying an endpoint on the network. If an endpoint is given, the robot host system deploys an SDC consumer which connects to the specified endpoint. The end-effector endpoint contains an SDC provider and supplies the needed transformation and mass definition. The data for end-effectors identified by ID is loaded from an internal database.

Finally, a third *VMD* “plan” includes metrics to receive a surgical plan from a suitable endpoint. The endpoint reference is set via a string metric and a *uniform resource identifier (URI)* can be specified to acquire the planning file. Another string metric is used to control the workflow by specifying the currently targeted trajectory.

## Technical limitations & improvements

### Surgical plan

For the accurate execution of a surgical plan, the robot system must be supplied with a dataset of all relevant trajectories, areas and other annotations to consider for path planning and the realization of movement constraints. The SDC standards do not include suitable means to transmit such a plan. It could be considered to serialize the surgical plan and transmit it to a *StringMetric*; however, such use is not compliant with the intended purpose of the *metric*. Furthermore, associated properties of the *metric* such as *Unit* or *Type* cannot be utilized to enable semantic interoperability intended by the standards.

For the exchange of a patient specific surgical plan, it is instead proposed to transmit the data outside of SDC via an open file format. Interoperability is not impaired via this method if the file format is also standardized and accessible. SDC communication can be used to set a *URI*, pointing to a file location either on a shared storage, a network directory or medical data archive. Furthermore, SDC is used to select the current workflow step in the surgical plan.

### Real-time communication

The present SDC standards cannot guarantee real-time data transmission with bounded latency. In the current version, TCP is used for transport and HTTP/2 is employed on the application layer. The traffic created by medical devices cannot be controlled efficiently with these technologies. Networks may become congested without any means to prioritize critical device data. This impacts the possible use-cases of surgical robots integrated via SDC; however, the device profile remains unchanged. Due to the lacking latency guarantees, the robot system cannot support synergistic control or dynamic movement compensation (control loop through the network). Instead, the robot can only operate as a semi-active positioning device. When the robot moves, the speed must be low since 10–100 ms of delay may be present in the safety unlock signal [14, 19].

A working group within OR.NET association is pursuing the creation of a co-standard named “real-time SDC” (RT-SDC). For the present work, it is assumed that bounded low-latency communication via SDC will be available in future. The proposed device profile will remain largely unchanged; however, the existing interface can support additional functionality. For instance, the robot can synergistically guide the surgeon toward the target trajectory in hands-on mode.

### Representation of matrices in the BICEPS data model

The largest technical hurdle for a real-world implementation of the proposed model is the lack of a dedicated *metric* type to represent a mathematical matrix. The transmission of 4 × 4 transformation matrices (or, alternatively, quaternions) is essential for applications including surgical navigation or robots. We considered three different alternatives to implement the lacking functionality with the BICEPS data model as-is:

1. Modeling as *DistributionSampleArray or RealTimeSampleArray.*
2. Modeling of a 4 × 4 matrix as 16 single *NumericMetrics.*
3. Modeling as a *StringMetric.*

All of these approaches are not suitable for real-world use: The *DistributionSampleArray* and *RealTimeSampleArray* are intended to represent series of temporally or stochastically related one-dimensional values, according to the *BICEPS* standard (11073–10207). Usage as a matrix is conflicting with the intended use and can lead to technical complications. For instance, the value series of a *RealTimeSampleArray* are being buffered and transmitted only in intervals in some IEEE 11073 software libraries. The second approach would create a large number of metrics in the *MdDescription*. Their relationship cannot be modeled within *BICEPS*. Furthermore, a single matrix update would trigger up to twelve single *SetValueOperations*, negatively impacting performance, and the “matrix” could be read by other devices while it is in an invalid state during the update.

For the current version standards, it is suggested to send a serialized matrix via *StringMetrics* as the “best” possible work-around. This way, a single metric can communicate the whole matrix, and further properties (such as row- or column-major orientation) can be included with the string. However, this approach still has major shortcomings which prevent its real-world use. For each consecutive date, the entries of the matrix must be converted to a decimal string representation which takes time and increases data size. In the string representation, each decimal digit takes up one byte. During the whole transfer, each number in the matrix is rounded twice, introducing noise to a control task.

It is proposed to define a new *MatrixMetric* type in *BICEPS* for dense matrices and vectors of arbitrary dimensions. The new type should be equipped with similar properties as the *NumericMetric* type, including a precision attribute and a physical unit within the 11073–10101 coding system.

### Coordinate systems in the operating room

The presented issues with coordinate transforms and the registration of multiple COS inspire a proposal to include a holistic description of reference frames in the operating room, including all medical devices and equipment. The *BICEPS* model currently includes a *LocationContext* and *BodySite* property for some entities. An addition to the model could include more precise position information for any object that can be localized within the operating room. Location providers beyond optical tracking are feasible, for instance through image detection of objects from the video stream of a documentation camera. Location data from additional medical devices could be used to realize new functionalities such as collision avoidance between OR table and X-ray C-arm.

Finally, an addition to the nomenclature system (11073–10101) could provide a better representation of links between SDC endpoints. The proposed robotic device profile of the present work includes three such links for the planning software, the surgical navigation and an optional active end-effector. These could be marked as such by specifying a new *CodedValue Unit* or *Type* describing *endpoint reference* strings and file locations (*URIs*). The former would also ease the identification of *Indicator metrics* and control associations for dynamic remote control as described Kasparick et al. [11].

## Conclusion

The proposed surgical robot interface and model cannot yet be adequately represented by the ISO IEEE 11073 SDC standards. The most crucial lacking features are a *metric* type for matrices and a mechanism to transmit a surgical plan. In addition, a possibility to send bounded low-latency data on the network would enable features which require a control loop over the network, such as synergistic hands-on control.

Yet, a generic robotic device profile can already be conceived and a large share of features can be realized with the current SDC standards. The model builds on existing classification schemes and incorporates concepts of related work where applicable. The ongoing trend of modularization in robotics can be represented in SDC. Modular components, such as active end-effectors, can implement their own provider endpoints and connect to other components.

In principle, the SDC standards are suitable to integrate surgical robotic systems into the connected OR. For the identified shortcomings, remedies were conceived which can be further discussed with technical research groups and used as a foundation for extended standards for robotic device profiles.
